# Affective touch in the context of development, oxytocin signaling, and autism

**DOI:** 10.3389/fpsyg.2022.967791

**Published:** 2022-11-23

**Authors:** Qin Li, Weihua Zhao, Keith M. Kendrick

**Affiliations:** ^1^School of Foreign Language, Chengdu University of Traditional Chinese Medicine, Chengdu, China; ^2^Ministry of Education, Key Laboratory for Neuroinformation, The Clinical Hospital of Chengdu Brain Science Institute, University of Electronic Science and Technology of China, Chengdu, China

**Keywords:** oxytocin, affective touch, autism (ASD), brain development, massage

## Abstract

Touch represents one of our most important senses throughout life and particularly in the context of our social and emotional experiences. In this review, we draw on research on touch processing from both animal models and humans. Firstly, we briefly describe the cutaneous touch receptors and neural processing of both affective and discriminative touch. We then outline how our sense of touch develops and summarize increasing evidence demonstrating how essential early tactile stimulation is for the development of brain and behavior, with a particular focus on effects of tactile stimulation in infant animals and pediatric massage and Kangaroo care in human infants. Next, the potential mechanisms whereby early tactile stimulation influences both brain and behavioral development are discussed, focusing on its ability to promote neural plasticity changes and brain interhemispheric communication, development of social behavior and bonding, and reward sensitivity through modulation of growth factor, oxytocin, and opioid signaling. Finally, we consider the implications of evidence for atypical responses to touch in neurodevelopmental disorders such as autism spectrum disorder and discuss existing evidence and future priorities for establishing potential beneficial effects of interventions using massage or pharmacological treatments targeting oxytocin or other neurochemical systems.

## Introduction

We are exquisitely sensitive to touch and being touched by or touching others in consensual contexts is highly pleasurable and of importance for the formation and maintenance of social and romantic bonds (Dunbar, 2010; Kreuder et al., 2017). Our ability to discriminate between self- and other-administered touch may also play a crucial role in developing our sense of self (Cioffi et al., 2014; Braun et al., 2018). However, in neurodevelopmental disorders, such as autism spectrum disorder (ASD), primarily characterized by problems with social communication and interaction and repetitive and restrictive behavior, receiving touch is often perceived and experienced as unpleasant or aversive and individuals can have hyper- or hypo-sensitivity to other sensory stimulation (Kanner, 1943; Lord et al., 2018). The current review aims to synthesize what we know about how affective tactile stimuli are detected and processed, the mechanisms whereby they can act to influence brain and behavioral development and how these may provide important future therapeutic opportunities in neurodevelopmental disorders. The review therefore firstly focuses on describing the way touch is perceived and processed and then considers its potential role in shaping brain and behavioral development, with a particular emphasis on the beneficial effects of pediatric massage and Kangaroo care in humans. The roles of specific neurochemical systems responsive to tactile stimulation in mediating key neural plasticity changes and social and cognitive development are then discussed, with particular emphasis on a pivotal role for the neuropeptide oxytocin. Finally, we discuss possible therapeutic effects of touch-based interventions for neurodevelopmental disorders such as ASD, Down syndrome, and cerebral palsy.

## Touch receptors and neural processing

Our sense of touch follows from stimulation of low-threshold afferent cutaneous fibers which convey distinct sensory/perceptual qualities *via* projections to different stimulus-specific neurons (McGlone et al., 2014; Croy et al., 2022). Touch can engage both fast conducting thick myelinated A-β afferents sub-serving touch discrimination in terms of pressure, vibration, and texture and thin unmyelinated slow-conducting ones including C-touch afferent fibers (CT-fibers) sub-serving affective aspects of touch (Björnsdotter et al., 2010; McGlone et al., 2012, 2014; Takahashi et al., 2015). CT-fibers are primarily present in hairy, as opposed to glabrous (palm and plantar foot regions) skin, respond selectively to slow, gentle, caress-like mechanical stroking (Ackerley et al., 2014; McGlone et al., 2014), and are associated with perceived pleasantness of social affective touch (Essick et al., 2010; Pawling et al., 2017). Activated CT-fibers can also reduce the effects of painful stimuli (Liljencrantz et al., 2013; Gursul et al., 2018).

CT-fibers may have evolved to sub-serve a homeostatic, protective, and emotional role (Croy et al., 2022; Morrison, 2022) and project *via* the dorsal horn and spino-thalamic tract primarily to the posterior insula cortex, whereas A-β fiber projections primarily target the somatosensory cortices (Olausson et al., 2002; McGlone et al., 2014; Croy et al., 2022). The posterior insular cortex plays a critical integrative role between somatosensory inputs from the skin and visceral processes as well as from nociceptors encoding pain (Morrison, 2022). Stimulation of CT-afferents influences the parasympathetic nervous system to reduce heart rate and increase heart-rate variability as well as reduce pain responses. CT-fiber afferent projections can subsequently activate brain reward regions such as the orbitofrontal cortex as well as the anterior cingulate and anterior insula and the superior temporal sulcus which are indispensable for social and affective processing (Olausson et al., 2010; Gordon et al., 2013; Björnsdotter et al., 2014; Croy et al., 2016; Morrison, 2016; Li et al., 2019). Not only do these regions respond to actual experience of touch but also when it is observed or imagined, with many being part of the brain “mirror” neuron system (Meltzoff et al., 2017; Chivukula et al., 2021).

Touch processing has both bottom-up and top-down components, with the latter contributing to modulation of responses to interpersonal touch. Thus, the same pattern of slow stroking of the skin can be perceived as more or less pleasant dependent upon who is delivering the touch (Scheele et al., 2014). Even young infants can show different parasympathetic responses to the same CT-fiber optimal touch by a stranger as opposed to their mother (Aguirre et al., 2019). These top-down processes involve frontal networks processing social salience, such as the cingulate and insula cortices which influence the ability of CT-fiber directed touch to activate brain reward systems (Scheele et al., 2014), and may also reflect cognitive contributions resulting from cross-hemispheric processing of touch stimuli. Thus, reinforcement learning about the salience of individuals who do the touching for social and calming purposes plays an important role in determining social touch preferences and forming social bonds.

## Brain and behavioral development and the influence of tactile stimulation

The somatosensory system is the first sensory system to develop prenatally, starting around 7–8 weeks of pregnancy, and is fully developed apart from some brain regions, such as the insula (Slater et al., 2006; Jönsson et al., 2018) and superior temporal sulcus (Miguel et al., 2019) involved in social cognition, by 32 weeks. Babies have a fine covering of soft “Lanugo” hair which grows from 17 to 26 weeks of gestation and may promote sensitivity to affective touch through activation of CT-fibers (Domagala et al., 2017). Possibly, the rhythmic stimulation of the CT-fiber system due to amniotic fluid movement promotes optimal calming effects on infants *in utero* and explains their preference for rhythmic rocking motion after birth (Bystrova, 2009). Abdominal stroking by the mother during pregnancy may also help promote bonding (Marx and Nagy, 2015). In terms of maturation of the processing of CT-fiber touch in the brain, this occurs particularly during the third trimester with parasympathetic effects on reducing heart rate reported in newborns (Van Puyvelde et al., 2019) and preterm infants (Manzotti et al., 2019) and stroking associated pain relief (Gursul et al., 2018). Touch-evoked responses in brain regions involved in regulating social cognition may primarily occur after birth, with the posterior insula becoming engaged around 2–3 months of age (Slater et al., 2006; Jönsson et al., 2018) and the superior temporal sulcus at around 12 months (Miguel et al., 2019).

Cutaneous projections from different parts of the body to the brain are somatotopically organized in the contralateral somatosensory cortex (i.e., left side of the body sends projections to right side of the brain and vice versa) even in preterm infants (Allievi et al., 2016). Functional connectivity between the two hemispheres also first develops prenatally (from 24 to 39 weeks of pregnancy, Thomason et al., 2013) and there is evidence for both contralateral and ipsilateral somatosensory cortex responses to touching of the body in newborn infants (Kusaka et al., 2011). It has been argued that discriminative and affective touch stimuli become increasingly bilaterally represented during development even at the earliest levels of processing (i.e., the somatosensory and insula cortices), and this is as a consequence of interhemispheric (transcallosal) connections rather than separate contralateral and ipsilateral ones (Genna et al., 2018; Tamé et al., 2019). Bilateral representation of touch may also have different developmental time-courses for different regions of the body, with regions such as the lips, but not hand or foot, showing it in 2-month-old infants (Meltzoff et al., 2017). A consequence of this bilateral representation of touch is that unilateral damage in the brain following stroke can result in loss of tactile sensitivity on both sides of the body (Dupin et al., 2014; Tamé et al., 2019). This suggests that the touch processing system in the brain plays a potentially important role in the integration and synchronization of communication between the brain hemispheres contributing to development of haptic, motor, and cognitive functions. Indeed, neurodevelopmental disorders such as ASD and schizophrenia with impaired cognitive and motor function are characterized by reduced interhemispheric connectivity (see Yao et al., 2021).

## Evidence for importance of touch for brain and behavioral development in premature infants

Premature infants exhibit delayed developmental milestones in areas such as motor skills, cognition, and language (Guerra et al., 2014) and can have subsequent sensorimotor integration problems (Pinheiro et al., 2014). In animal models, massage of preterm infants accelerates maturation of both cortical electroencephalography and visual acuity (Guzzetta et al., 2009). In humans, pediatric massage is extensively used in neonatal intensive care units (NICUs) and has consistently been shown to produce beneficial effects in premature infants, most notably for promoting growth and health and earlier discharge from NICUs (see Field, 2002, 2019; Badr et al., 2015; Niemi, 2017; Liao et al., 2021). Additionally, studies have reported positive results of massage on immune system (Karamian et al., 2022) as well as on cognitive and visual function and visuomotor integration (Guzzetta et al., 2009; Fontana et al., 2020; Campbell and Jacobs, 2021) and on the strength of mother–infant bonding (Shoghi et al., 2018). Currently, many different forms of pediatric massage have been used across cultures. In general, these tend to be whole-body stroking, medium pressure massages administered daily or several times a week for 15 min and either with or without oil (see Badr et al., 2015; Field, 2019; Chaturvedi et al., 2021).

Other research on preterm human infants has reported beneficial effects of skin-to-skin contact and touching during so called: “Kangaroo care.” Kangaroo care involves a naked infant carried regularly in an upright prone position against the bare chest of a parent for 1–3 h at a time. As with pediatric massage, it provides extensive tactile stimulation for both infant and mother and there is similar evidence that it can improve growth, responses to stress and pain, immune responses, and development of cognitive and sensorimotor function, as well as having advantageous effects on mutual bonding between parent and infant and parental mood (Feldman et al., 2001; Jefferies, 2012).

## Research on brain and behavioral effects of early post-natal touch in full-term infants

Extensive research in rats has established that tactile stimulation of neonates either from mothers, in the form of licking and grooming (Meaney, 2001, 2010), or even administered artificially (Hellstrom et al., 2012), produces lasting effects on their subsequent social and anxiety and cognitive behaviors which can be passed on to the next generation. An epigenetic mechanism was identified for this effect influencing glucocorticoid expression in the hippocampus as well as on the oxytocin system which plays a major role in both social and maternal behavior and on anxiety as well as cognitive function (Meaney, 2010; Kundakovic and Champagne, 2015). Recent research in mice has shown that short periods of stroking the back region of pups has profound effects on the responses and development of the brain oxytocin system as well as on social behavior and sensitivity to reward (Yu et al., 2022). Together, these animal model studies have particularly implicated touch-evoked effects on the brain oxytocin system as being important for mediating developmental changes (see below for further discussion).

In monkey infants additional handling by humans in the early post-natal period increases exploratory, social, and cognitive development (Simpson et al., 2019). In full-term human infants, a number of studies have reported beneficial effects of pediatric massage on fine and gross motor skills, social and personal behavior, bonding and adaptive behavior, and psychomotor development, although studies to date are in need of more extensive replication (Wahyutami et al., 2010; Bennett et al., 2013; Perez et al., 2015). A recent study on full-term infants has reported that only a few minutes of gentle stroking by either parent has positive effects on cardio-respiratory function in full-term infants (Van Puyvelde et al., 2019). Furthermore, another study reported that skin-to-skin contact during Kangaroo care in full-term infants promoted greater social development over a 9-year period in a longitudinal study including 90 mother–child dyads (Bigelow and Power, 2020). Further indirect evidence for the significance of receiving affective touch during early life comes from some early studies on individuals raised in institutionalized environments who exhibit cognitive and visuomotor integration impairments (Frank et al., 1996; Cermak and Groza, 1998). Furthermore, if such individuals receive an additional 20 min of tactile stimulation per day for 10 weeks as infants, they have higher subsequent scores on cognitive and behavioral developmental assessments (Casler, 1965).

## The influence of touch on growth factor, oxytocin, and opioid signaling

While tactile stimuli can potentially affect a wide range of neurochemical systems to influence brain and social development, the greatest focus has been on ones which are involved in neural plasticity and growth (growth factors), reward processing, and social behavior (opioids and oxytocin; see also Carozza and Leong, 2021).

In animal model studies, touch stimuli administered to infant rodents increase brain concentrations of insulin growth factor-1 (IGF-1) associated with functional maturation effects on visual processing and this could be prevented by administering an IGF-1 antagonist (Guzzetta et al., 2009). Touch stimuli in the form of maternal licking in rodents also influence nerve growth factor signaling (Hellstrom et al., 2012), and in human infants, massage increases peripheral concentrations of both insulin and IGF-1 which are associated with general body growth (Field et al., 2008; Field, 2019). Such increases in growth factor release within the brain will also promote important neural plasticity changes underlying brain and behavioral development.

The most extensively researched neurochemical system which responds to social touch and massage is that of the neuropeptide oxytocin produced by neurons in the supraoptic (SON) and paraventricular (PVN) nuclei of the hypothalamus and with receptors expressed widely in the brain (Quintana et al., 2019). Large magnocellular neurons from the SON and the PVN project to the posterior pituitary, releasing oxytocin into the circulation to act on its receptors in a number of peripheral organs including the adrenal gland, breast, cardiovascular and gastrointestinal systems, uterus, ovaries, testes where it primarily acts to influence their functions by contracting smooth muscle. These organs also have cells which can secrete oxytocin, including endothelial cells and keratinocytes in the epidermal layer of the skin (Deing et al., 2013). There is widespread expression of the oxytocin receptor in the brain (Jurek and Neumann, 2018; Quintana et al., 2019) and oxytocin can be released within the brain from cell dendrites and axonal projections from magnocellular neurons, or from smaller parvocellular neurons which do not influence release from the posterior pituitary. These regions include frontal, cingulate, and insula cortices, basal ganglia, limbic system (amygdala, hippocampus, and septum), midbrain, brainstem, and spinal cord (see Liao et al., 2020), suggesting that oxytocin neurons can have widespread influence on behavioral, physiological, and neuroendocrine functions. Some parvocellular PVN neurons additionally project to the anterior pituitary where they can influence release of stress hormones such as adrenocorticotrophic hormone (see Grinevich and Stoop, 2018; Jurek and Neumann, 2018; Uvnäs-Moberg and Petersson, 2022 for reviews of the oxytocin system).

Tactile stimulation during social interactions, skin-to-skin contact, or massage is considered an important mediator of oxytocin release. Activation of CT-fiber cutaneous fibers is considered to be of the most importance although other C-fibers and myelinated A-β fibers could also be involved (Uvnäs-Moberg et al., 2015; Walker et al., 2017; Takahashi, 2021; Uvnäs-Moberg and Petersson, 2022). Oxytocin release can play a key role in modulating social interactions, bonding and affective processing (Kendrick et al., 2017) as well as influencing a range of physiological and endocrine functions, particularly in terms of reducing stress and pain through PVN projections to the brainstem and modulation of the hypothalamo-pituitary–adrenal stress axis (see Bharadwaj et al., 2021; Carozza and Leong, 2021; Takahashi, 2021; Uvnäs-Moberg and Petersson, 2022). The PVN oxytocin neurons receive projections from the insula cortex (McGlone et al., 2014) and it is likely that this is the main pathway by which activation of CT-fibers during affective touch initially influences the brain oxytocin system. A recent animal study has shown that a population of PVN oxytocin neurons responds to social and non-social tactile stimulation primarily targeting CT-fibers and co-ordinates interactions with the more extensive magnocellular system, resulting in brain-wide activation of the oxytocin projection system and oxytocin release (see Tang et al., 2020). Indeed, tactile stimulation is consistently reported to increase peripheral oxytocin release in animal models (Stock and Uvnäs-Moberg, 1988; Uvnäs-Moberg and Petersson, 2010; Mitsui et al., 2011; Schneiderman et al., 2012; Crockford et al., 2013; Uvnäs-Moberg et al., 2015; Vittner et al., 2018). Furthermore, the effects of tactile stimulation on enhancing the social and behavioral development of rodents are also directly associated with facilitation of oxytocin signaling in the brain (Yu et al., 2022).

In humans, both post-partum mothers and their infants have increased peripheral oxytocin concentrations during skin-to-skin contact, such as would occur during Kangaroo care (Matthiesen et al., 2001; Uvnäs-Moberg and Petersson, 2010; Uvnäs-Moberg et al., 2015) and this may serve to reduce stress and facilitate mutual bonding between mother and infant (Uvnäs-Moberg and Petersson, 2010). “Warm touch” between couples and affectionate touch during early stage of romantic relationships also increases peripheral oxytocin concentrations (Light et al., 2005; Holt-Lunstad et al., 2008; Schneiderman et al., 2012). Slow, moderate pressure massage in adult humans facilitates peripheral oxytocin release (Morhenn et al., 2012; Li et al., 2019), and foot massage administered by hand, but not by machine, increases activity in key brain regions involved in pleasurable (orbitofrontal cortex) and social cognition (superior temporal sulcus) aspects of affective touch, although not in the somatosensory cortex (Li et al., 2019).

A number of studies have investigated the effects of intranasal administration of oxytocin on brain and behavioral responses to affective touch or massage. Thus, intranasal oxytocin can increase both the perceived pleasantness of touch administered *via* different materials and activation of the orbitofrontal cortex independent of touch valence (Chen et al., 2020a). Intranasal oxytocin also augments perceived pleasantness of hand- but not machine-administered massage as well as increasing responses in the majority of regions in the social brain, including those involved in attention, social cognition, reward, and emotional responses. Interestingly, effects on neural responses occur with both real and imagined massage further emphasizing evidence that the affective touch system responds in a similar way to experienced and observed touch (Chen et al., 2020b). In respect of the potential importance of early tactile stimulation on the development of optimal communication between the two brain hemispheres, intranasal oxytocin also strengthens effective interhemispheric connectivity between many regions of the social brain (Jiang et al., 2021). Finally, intranasal oxytocin has been reported to have pain-relieving effects (Bharadwaj et al., 2021) and thus potentially some of the well-established nociceptive effects of tactile stimulation may be mediated *via* modulation of both central and peripheral oxytocin release.

An important aspect of the influence of oxytocin on rewarding effects of tactile processing is through its established interactions with both dopaminergic and opioid systems also intimately involved with both social bonds and brain reward systems. The opioid system in particular is important for formation of social bonds (Machin and Dunbar, 2011) and social touch modulates μ-receptor activity in the insular cortex and frontal and striatal reward systems (Nummenmaa et al., 2016). As already discussed, the insular cortex plays a key role in responding to CT-fiber-mediated tactile stimuli and may be a key region where interactions between oxytocin, opioid, and dopaminergic neurochemical systems occur and subsequently influence social attention and reward systems (Loth and Donaldson, 2021).

In terms of person-specific effects on affective touch, intranasal oxytocin can increase perceived pleasantness of social touch applied to the leg of male subjects as well as greater activation of the orbitofrontal cortex and insula, but only when subjects thought touch was administered by a female (Scheele et al., 2014). Similarly, oxytocin increased likeability of touch in subjects when they thought they had been touched by their partner but not by an unfamiliar person of the opposite sex (Kreuder et al., 2017). Thus, there is both evidence for oxytocin administration producing general effects on perceived pleasure of CT-fiber targeted touch and on brain attention and reward processing networks but also linking them specifically to the identity of preferred individuals administering the touch. Overall, therefore, oxytocin may potentially influence both top-down and bottom-up aspects of touch processing and play an important role in modulating neural circuitry involved in both attentional and rewarding aspects of touch.

**Figure 1:**
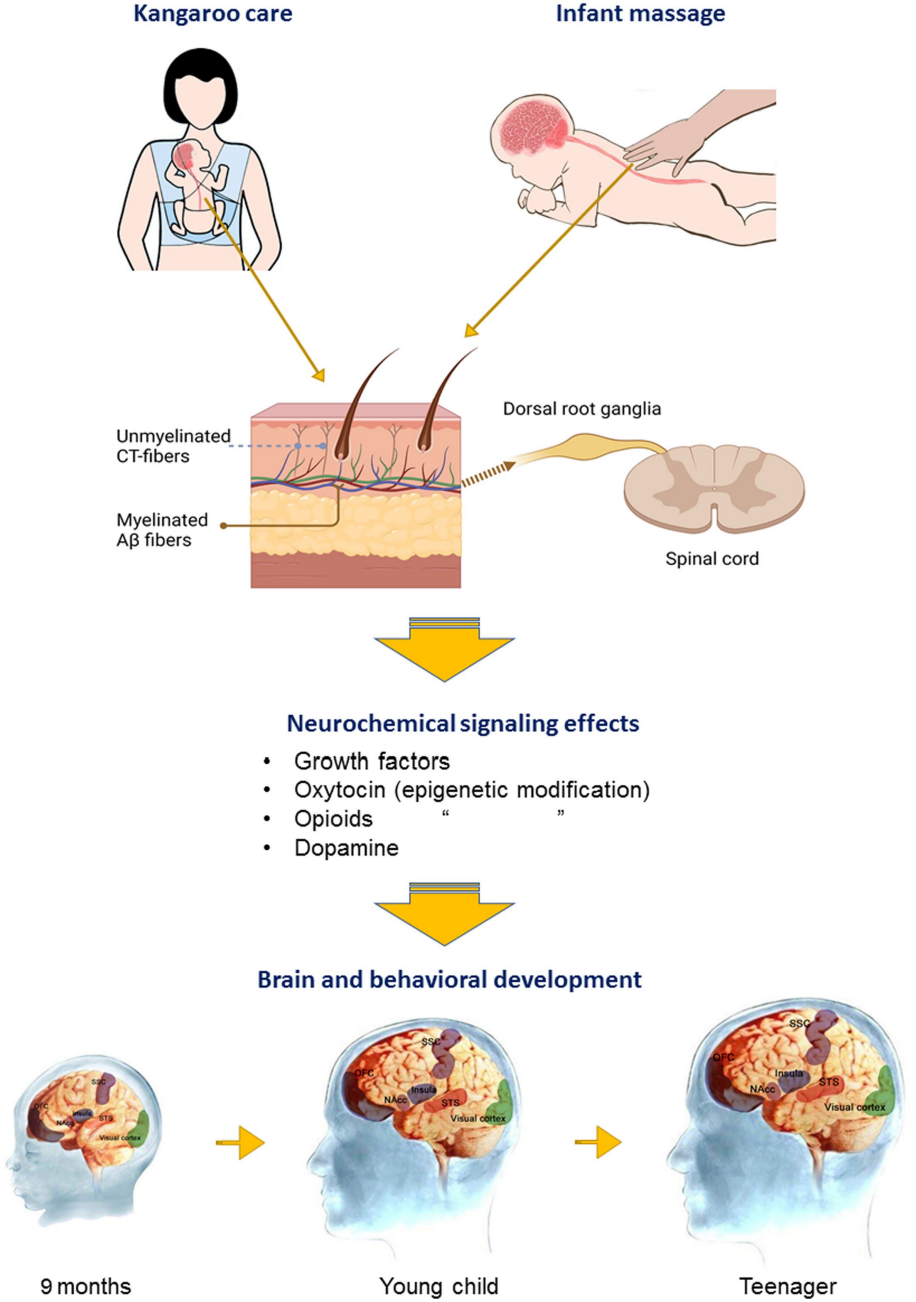
Influence of affective touch on brain development. Schematic illustrates effects of either parental touch (Kangaroo touch) or pediatric massage on cutaneous fibers for affective (CT-fibers) or discriminative touch (Aβ) and projections to the brain *via* the dorsal route ganglia and spinal cord. Affective touch in particular promotes brain development and cognitive and social behavior *via* stimulation of growth factors, and oxytocin, opioid, and dopamine release and signaling and epigenetic modification. These neurochemical systems help to mediate developmental changes *via* neural plasticity and integration of brain circuitry sub-serving sensory (visual cortex, somatosensory cortex—SSC), salience (insula cortex), reward (orbitofrontal cortex, OFC and nucleus accumbens, NAcc), and social cognition (superior temporal cortex, STS) processing. Responses of the STS to touch are not fully developed until around 12 months of age.

Both oxytocin (Puglia et al., 2018; Perkeybile et al., 2019) and opioid (Vucetic et al., 2011; Browne et al., 2020) receptor expression undergo either upregulation or downregulation *via* epigenetic modification and experience of tactile stimulation may influence this. Oxytocin also promotes neural plasticity changes in sensory, attention, and social processing brain regions (Froemke and Young, 2021). There are therefore several mechanisms whereby early experience of tactile stimuli may act to either facilitate or impair the optimal integration effects of tactile stimuli on brain and social development and reward *via* modulation of both oxytocin and opioid systems, as well as *via* enhancing growth factor signaling (see Figure 1).

## Touch and neurodevelopmental disorders

Hypo- or hyper-sensitivity to sensory stimuli is common in ASD and is now one of the features incorporated into DSM-V. Up to 90% of individuals with ASD have atypical responses to touch (see Espenhahn et al., 2022) with soft touch often perceived as aversive/painful in hyper-sensitive individuals or without any significance in hypo-sensitive ones. There is debate as to whether these two extremes have similar or different underlying mechanisms, since while they can be associated with either increased or decreased responses in CT-fibers and the somatosensory cortex, they have similar patterns of reduced responses in brain regions involved in social cognition and motivation, such as the superior temporal sulcus and orbitofrontal cortex (Kaiser et al., 2016). Indeed, both the degrees of hyper- or hypo-sensitivity to touch are associated with severity of social dysfunction (see Mikkelsen et al., 2018; Thye et al., 2018). Although ASD is contributed largely by genetic factors, premature birth is one of the main experiential ones which can increase its prevalence by up to 4-times (Crump et al., 2021). Furthermore, children with ASD have consistently been shown to have reduced peripheral concentrations of oxytocin (John and Jaeggi, 2021; Moerkerke et al., 2021). Additionally, several studies have reported increased epigenetic methylation of the oxytocin receptor, with resultant reduced mRNA expression associated with the severity of symptoms and altered functional connectivity in brain regions involved in the control of theory of mind, social attention, and reward processing (Gregory et al., 2009; Puglia et al., 2018; Andari et al., 2020).

The observations summarized above suggest that a touch-based therapy, and/or one designed to enhance oxytocin signaling, could have beneficial effects on reducing ASD symptoms and additionally atypical sensory responses, by influencing brain and social development. To date, only one small study has reported that massage can increase peripheral concentrations of oxytocin in autistic boys (Tsuji et al., 2015), similar to typically developing individuals (Li et al., 2019). A number of small-scale studies have reported some positive effects of massage-based interventions on ASD symptoms and cognitive function, and in reducing atypical sensory responses, although there is a need for these to be confirmed by large-scale randomized controlled trials (see Weitlauf et al., 2017). Notably, two studies by the same group have reported promising effects in 3–6 year-old autistic children using a number of sensorimotor environmental enrichments, including massage, on improved symptoms, social and cognitive development, and sensitivity to sensory stimuli (Woo and Leon, 2013; Woo et al., 2015). There are currently no brain imaging studies investigating effects of such massage interventions on altered neural development, interhemispheric connectivity, and responses to touch in ASD and this must be a priority for future studies. The potential for a massage intervention in premature infants to reduce the proportion of “at risk” infants from subsequently developing ASD has also yet to be assessed. In relation to the effects of oxytocin-based treatment interventions in young children several recent clinical trials have reported that chronic intranasal oxytocin treatment can improve social symptoms (Yatawara et al., 2016; Parker et al., 2017; Le et al., 2022), although dose frequency and combining treatment with positive social interactions may be important (Le et al., 2022). To date, no oxytocin intervention trials in ASD have assessed whether it alters either neural or behavioral responses to tactile stimuli and future studies will need to address this. In terms of other developmental disorders, pediatric massage has been reported to facilitate psychomotor development in infants with Down syndrome or cerebral palsy (Silva et al., 2012; Purpura et al., 2014; Pinero-Pinto et al., 2020), although again further large-scale controlled trials are needed to confirm this.

Overall, there is increasing evidence for the importance of tactile stimuli *via* affective CT-fiber cutaneous receptors for typical development of both brain and behavior and that many of its effects are likely mediated *via* facilitation of growth factor, oxytocin, and opioid systems. However, more animal model and human-based research is required to fully establish optimal stimuli, the mechanisms involved, and the potential for development of therapeutic interventions in neurodevelopmental disorders.

## Author contributions

QL, WZ, and KK conceived the idea for the review and wrote it. All authors contributed to the article and approved the submitted version.

## Funding

This work was supported by Key Technological Projects of Guangdong Province “Development of New Tools for Diagnosis and Treatment of Autism” grant no. 2018B030335001.

## Conflict of interest

The authors declare that the research was conducted in the absence of any commercial or financial relationships that could be construed as a potential conflict of interest.

## Publisher’s note

All claims expressed in this article are solely those of the authors and do not necessarily represent those of their affiliated organizations, or those of the publisher, the editors and the reviewers. Any product that may be evaluated in this article, or claim that may be made by its manufacturer, is not guaranteed or endorsed by the publisher.
